# Improvements in self-efficacy and attachment security during the psychodrama group process among young women: A mix-methods study

**DOI:** 10.1192/j.eurpsy.2025.2158

**Published:** 2025-08-26

**Authors:** I. G. Yilmaz-Karaman, N. Kalkan-Oguzhanoglu, O. Öner, T. Gunduz

**Affiliations:** 1 Psychiatry, Eskişehir Osmangazi University, Faculty of Medicine, Eskişehir; 2 Psychiatry, Pamukkale University, Faculty of Medicine, Denizli; 3 Bergama State Hospital, İzmir, Türkiye

## Abstract

**Introduction:**

Mental illnesses are more common in women than men, and common illnesses such as anxiety and depression often begin in youth. Young women are disadvantaged both in terms of developing mental illnesses and in terms of gender-based discrimination. In this respect, protective, preventive, and empowering interventions targeting young women can be a good investment in terms of protecting their mental health. The youth period is the focus of protective and preventive interventions due to its nature as the initial period of frequently seen mental illnesses. Empowerment can reduce vulnerability during this period.

**Objectives:**

This study aimed to examine quantitatively and qualitatively whether the self-efficacy and attachment dimensions of the participating young women changed as a result of an empowerment-themed psychodrama group therapy.

**Methods:**

The study has a longitudinal design with mix methods. After the local ethics committee approval, an announcement was made online, and the participants were included in the study after face-to-face meetings. Young women between 18 and 25 years who do not have a current psychiatric diagnosis, without suicide attempts and alcohol or substance use disorder, were recruited. Seventeen psychodrama sessions, each lasting three hours, were conducted. Role reversal, pairing, and mirror techniques were mainly used in the sessions. Warm-up games and group games were selected following the theme of empowerment. The General Self-Efficacy Scale (GSS) and Experiences in Close Relationships- Revised (ECR-R) were applied to the group members before starting the psychodrama sessions and after the sessions ended. In addition, the group members’ processes were investigated using qualitative methods after the sessions had ended.

**Results:**

Thirteen young women completed the psychodrama group sessions without more than 20% absence. Statistically significant increases were observed in the initiation (z=-2.310 p=0.021), resilience (z=-1.973 p=0.049), maintenance effort sub-dimensions (z=-2.355 p=0.019), and GSS total score (z=-2.357 p=0.018) of general self-efficacy after the intervention. When the sub-dimensions of ECR-R were evaluated, statistically significant decreases were observed in both avoidant attachment (z=-2.831 p=0.005) and anxious attachment (z=-2.864 p=0.004). Qualitative analysis showed themes of self-confidence, modeling, and universality.

**Conclusions:**

Psychodrama group psychotherapy is suitable for developing self-efficacy and increasing secure attachment in young women as a preventive psychiatric intervention.

**Disclosure of Interest:**

None Declared

